# Intra- and peritumoral radiomics for predicting equivocal HER2 status of breast cancer on contrast-enhanced mammography

**DOI:** 10.3389/fonc.2026.1807530

**Published:** 2026-04-29

**Authors:** Cong Xu, Juan Qiu, Shijie Zhang, Yuqian Chen, Xiaodong Wang, Haicheng Zhang, Qi Wang, Tongpeng Chu, Ziyin Li, Peng Lu, Haizhu Xie, Heng Ma, Nina Qu, Ning Mao, Jing Liu, Runpeng Chen, Jing Gao

**Affiliations:** 1 Physical Examination Center, Yantai Yuhuangding Hospital, Qingdao University, Yantai, Shandong, China; 2 Department of Breast, Guilin Municipal Hospital of Traditional Chinese Medicine, Guilin, Guangxi, China; 3 Department of Radiology, Huangshi Central Hospital, Affiliated Hospital of Hubei Polytechnic University, Huangshi, Hubei, China; 4 Big Data and Artificial Intelligence Laboratory, Yantai Yuhuangding Hospital, Qingdao University, Yantai, Shandong, China; 5 Department of Radiology, Yantai Yuhuangding Hospital, Qingdao University, Yantai, Shandong, China; 6 Shandong Provincial Key Medical and Health Laboratory of Intelligent Diagnosis and Treatment for Women’s Diseases, Yantai Yuhuangding Hospital, Yantai, Shandong, China; 7 School of Life Sciences, Ludong University, Yantai, Shandong, China; 8 Department of Ultrasound, Yantai Yuhuangding Hospital, Qingdao University, Yantai, Shandong, China; 9 Department of Interventional Operating Room, Yantaishan Hospital, Yantai, Shandong, China; 10 Shanghai Tenth People’s Hospital, School of Medicine, Tongji University, Shanghai, China

**Keywords:** breast cancer, contrast-enhanced mammography, equivocal HER2 status, intra- and peritumoral radiomics, nomogram

## Abstract

**Objective:**

Identification of Human epidermal growth factor receptor 2 (HER2) status is significant for the treatment and prognosis of breast cancer patients. The study aimed to evaluate the equivocal HER2 (IHC 2+) status of breast cancer using intra- and peritumoral radiomics features of contrast-enhanced mammography (CEM).

**Methods:**

A total of 131 breast cancer patients with equivocal HER2 (IHC 2+) status of breast cancer were enrolled in the study and divided into training (n=84), internal test (n=22) and prospective test (n=25) cohorts. Radiomics features were extracted from intratumoral and peritumoral regions on CEM and were selected using low variance and least absolute shrinkage and selection operator regression (LASSO). Five radiomics signatures were established based on different intratumoral and peritumoral regions. The nomogram was constructed using the selected signatures and clinical factors by logistic regression analysis. Its predictive performance was compared with the radiomics model and the clinical model. The area under the receiver operator characteristic curve (AUC), sensitivity, specificity, accuracy, the calibration curve, and decision curve analysis (DCA) were used to evaluate predictive performance of the models.

**Results:**

The intratumoral signature, 5mm-peritumoral signature, and tumor diameter were used to establish nomogram. Compared to the radiomics model and the clinical model, the nomogram achieved optimal predictive performance, with an AUC of 0.893 in the internal test cohort and an AUC of 0.840 in the prospective test cohort. The calibration curves and DCA showed favorable predictive performance of the nomogram.

**Conclusions:**

The nomogram incorporated the intratumoral and peritumoral radiomics signatures of CEM and clinical risk variables has the potential to predict equivocal HER2 (IHC 2+) status of breast cancer preoperatively.

## Introduction

Human epidermal growth factor receptor 2 (HER2)-positive breast cancer is mainly an aggressive subtype of breast cancer that accounts for about 15-20% of all breast cancers (1). The HER2 gene is an independent prognostic factor for breast cancer recurrence and survival, a series of studies confirmed that the overexpression and amplification of the HER2 gene are closely associated with the effectiveness of breast cancer treatment (2–4). Randomized clinical trials have demonstrated that adding anti-HER2-positive therapy to the treatment of HER2-positive breast cancer patients is an important factor for improving patient outcomes, which were reflected in an increase in the pathological complete response (pCR) rate, overall survival rate, and disease-free survival rate, as well as a reduction in the risk of disease recurrence or death (5–7). Therefore, the timely identification of HER2 status is of paramount importance for guiding subsequent treatment for guiding subsequent treatment. Immunohistochemical (IHC) detection of protein overexpression and fluorescence *in situ* hybridization (FISH) analysis of HER2 gene amplification are two principal methods for determining HER2 status (8). HER2 positivity is defined either by protein overexpression as defined by IHC3+ or equivocal protein expression (IHC2+) with evidence of HER2 gene amplification (9). IHC 2+ was equivocal and required FISH to ascertain whether it was amplified (10). In the clinic, the IHC test is relatively straightforward. However, additional analysis, such as FISH, undoubtedly results in time-consuming procedures and associated additional costs. It also requires specialized equipment and technical expertise. Consequently, a convenient and non-invasive modality to predict equivocal HER2 (IHC 2+) status is required.

Imaging is an essential tool in medical science and is routinely used in clinical practice for tumor detection and treatment guidance (11). Contrast-enhanced mammography (CEM) represents a cutting-edge technique that utilizes dual energy exposure, which has the advantage of low cost, time-saving, and acceptable tolerance in breast cancer patients. It could not only display the morphological characteristics, but also provide the blood supply information of the lesions. Moreover, CEM has higher sensitivity and specificity in the diagnosis of breast cancer than mammography and comparable to dynamic contrast-enhanced magnetic resonance imaging (DCE-MRI) (12–14). However, it is difficult to assess equivocal HER2 (IHC 2+) status using only the image features visible to the naked eye. Radiomics is a promising tool that can convert medical images into mineable data by extracting high-throughput quantitative features (15). As a non-invasive and effective method, radiomics has been widely used to assess whole tumor heterogeneity (16), and some researches have attempted to use radiomics to predict HER2 status in breast cancer (17–19). However, these studies were conducted based on MRI or mammography, not CEM. Besides, the tumor microenvironment plays a role in the development and progression of breast cancer. Some literatures have explored the value of peritumoral radiomics in breast cancer diagnosis and prognosis (20–22). Empirical evidence implicates that the microenvironment may contain information that is relevant to the treatment of HER2-positive breast cancer (23, 24). However, the value of peritumor radiomics based on CEM in predicting equivocal HER2 (IHC2+) status is unclear.

Despite the rapid emergence of advanced imaging techniques and artificial intelligence–based diagnostic tools in breast cancer, a substantial gap persists between technological innovation and clinical implementation. Recent evidence highlighted that while novel modalities, such as hyperspectral imaging, could offer promising diagnostic capabilities, their translation into routine practice was often hindered by issues related to standardization, interpretability, and integration into clinical workflows (25). Similarly, computer-aided detection systems have demonstrated variable performance across different imaging platforms, with trade-offs between sensitivity and specificity that limit their standalone utility in complex diagnostic tasks such as equivocal HER2 status determination (26). In this context, CEM represents a pragmatic alternative as it is already integrated into clinical practice. This study aims to extract radiomics features based on CEM and combine them with clinical characteristics to construct nomogram. Which focuses on clinical applicability, the nomogram would provide a non-invasive and easily generalizable solution for preoperatively predicting equivocal HER2 (IHC 2+) status, thereby helping to reduce reliance on FISH testing, thus assisting in clinical practice.

## Methods

### Study population

Ethical approval was obtained for retrospective study, and the requirement for patient informed consent requirement was waived. A prospective study was approved by the institutional ethics committee of the hospital. Written informed consents were obtained from patients whose CEM images were prospectively collected. (Clinical trial number: not applicable).

We retrospectively screened the CEM images between January 2021 and January 2022 in the institution from the picture archiving and communication system. The inclusion criteria for the retrospective study were as follows: (1) breast cancer patients pathologically confirmed by biopsy or surgical specimens; (2) underwent CEM less than 2 weeks before surgery or other treatment options; (3) patients with a single lesion; and (4) HER2 score of 2+ verified by IHC and have the results of FISH. The exclusion criteria were as follows: (1) patients who underwent breast radiotherapy, chemotherapy, or hormone treatment before CEM examination; (2) incomplete clinical, pathological, IHC or FISH information of patients; and (3) poor image quality. A total of 106 female patients were ultimately included in this study and randomly divided into training cohort and internal test cohort at a ratio of 8:2. In addition, 25 patients were prospectively evaluated at our hospital from March 2022 to June 2022. For the prospective study, the inclusion criteria were as follows: (1) patients were confirmed by biopsy as breast cancer; (2) enrolled patients were planned to receive standard treatment or surgery in our hospital; (3) CEM examinations were available and performed within two weeks before surgery or other therapeutic regimes; and (4) HER2 score of 2+ verified by IHC and have the results of FISH. The exclusion criteria were as follows: (1) patients who had any previous history of cancer and treatment; (2) patients with incomplete clinical, pathological, IHC or FISH information; and (3) inadequate image quality or non-mass lesions which affected the observation and delineation. Information regarding the clinical characteristics of the patients, including age and other parameters, were obtained from the electronic medical record.

### CEM examination

All patients underwent CEM examination using a GE Senographe Essential mammography unit (GE Healthcare, Milwaukee, WI, USA). The contrast agent Omnipaque 300 (GE Healthcare, Inc., Princeton, NJ) was injected into the upper arm vein with the dose of 1.5 ml/kg and the injection flow rate of 3.0 ml/s. Two minutes after the contrast injection, images were obtained in the following order: craniocaudal (CC) and mediolateral oblique (MLO) views of the suspicious breast, and then CC and MLO views of the less suspicious breast. For each mammographic projection, a pair of high-energy (HE) and low-energy (LE) exposures were consecutively performed to obtain HE and LE images, and a recombined (RC) image was generated automatically from LE and HE images using a dual-energy weighted logarithmic subtraction technique (27).

### Pathological assessment

According to the recommendations by the American Society of Clinical Oncology (ASCO)/College of American Pathologists (CAP) guidelines, IHC analyses were performed to determine the expression levels of HER2 in each breast cancer patient. A HER2 staining intensity score of 3+ was considered positive, while a score of 0 or 1+ was considered negative. A HER2 staining intensity score of 2+, with confirmation of gene amplification by FISH, was also deemed positive HER2 (10, 28, 29). In this study, all pathology reports were reviewed by two breast pathologists with 7 and 10 years of experience, respectively. Disagreements between the two primary evaluators were resolved by a senior pathologist with 20 years of experience, whose assessment was considered final.

### Image segmentation

All radiologists who participated in the image segmentation were blinded to the clinical and histopathological information. A dedicated radiologist (Segmenter 1) with seven years’ experience of breast imaging annotated tumor boundaries as the intratumoral region (ITR) in the LE and RC images with standard CC view via the ITK-SNAP (version 3.8.0; www.itksnap.org) software. All the contours were reviewed by a radiologist with 15 years of experience. Meanwhile, 40 patients randomly selected from the training cohort were used to assess the consistency of the inter- and intra-observer segmentation. Another 2 two radiologists (Segmenter 2 and 3) with 8 and 10 years of experience, respectively, performed the segmentation work utilizing the same methods. Additionally, the radiologist (Segmenter 1) repeated the segmentation with an interval of two weeks. The Dice similarity coefficient (DSC) was used to evaluate the agreement both inter- and intra-observer segmentation. Average DSCs of 0.873 and 0.925 were achieved for inter- and intra-observer segmentation performances, respectively. Meanwhile, the largest diameters of the lesions were measured independently by the two radiologists in CC view images. The mean values were calculated as the final tumor diameter.

After the ITR mask was annotated, a morphologic operation of dilation was performed to capture the peritumoral regions (PTR) outside the tumor of 5 mm and 10 mm using Python (version 3.6.6). Specifically, the pixel spacing information, representing the physical size of each pixel, was first extracted from the original images. The images were then resampled to a uniform pixel spacing of 1 mm, so that physical distances of 5 mm and 10 mm corresponded to 5 and 10 pixels, respectively. Based on these converted pixel distances, circular structuring elements with radii of 5 and 10 pixels were constructed. Subsequently, two-dimensional morphological dilation was applied to the tumor segmentation mask using these structuring elements, yielding dilated regions that encompassed the tumor and its surrounding areas of 5 mm and 10 mm. In addition, the breast region was segmented from the background in the original images via Otsu thresholding. To avoid including non-breast tissues in the peritumoral regions, the dilated regions were constrained by the breast region mask whenever they extended beyond the breast parenchyma. Similarly, any portions of the dilated regions that exceeded the image boundaries were cropped to retain only the pixels within the valid image range (21). Additionally, we define the intra- and peritumoral region in the same ROI named as IPTR.

**Figure 1:**
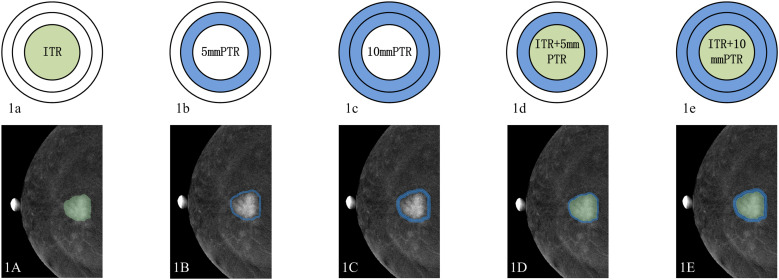
The sketch maps of different ROIs on RC-CC view of CEM image. **(1a, 1A)** ITR, intratumoral region; **(1b, 1B)** PTR5, 5-mm peritumoral region; **(1c, 1C)** PTR10, 10-mm peritumoral region; **(1d, 1D)** IPTR5: intratumoral region plus 5-mm peritumoral region; **(1e, 1E)** IPTR10, intratumoral region plus 10-mm peritumoral region.

Finally, for each lesion in each image, five ROIs, namely ITR, PTR5 (5-mm peritumoral region), PTR10 (10-mm peritumoral region), IPTR5 (intratumoral region + 5-mm peritumoral region) and IPTR10 (intratumoral region + 10-mm peritumoral region), were generated to extract radiomics features. Example segmentations are presented in **Figure 1**.

### Radiomics feature extraction

Image preprocessing was conducted by Python (version 3.6.6) before radiomics feature extraction. First, all images were resampled to a uniform pixel spacing of 1 mm pixel to ensure consistency in spatial resolution across different acquisitions. Subsequently, image pixel intensities were normalized by mapping the grayscale range uniformly to [0,255], thereby minimizing inter-image brightness variations. Finally, gray-level discretization was applied to the normalized images, the continuous intensity values were partitioned into fixed-width bins (bin width = 8, bin count = 32), and all pixels falling within a given bin were assigned the same gray level. This step reduces the sensitivity of texture feature computation to noise and enhances feature stability. All preprocessing steps were executed automatically to guarantee the reproducibility of the feature extraction process. Radiomics function RadiomicsFeatureExtractor toolkit provided by Pyradiomics (30) was used to perform radiomics feature extraction. A total of 1316 quantitative radiomics features were extracted from each ROI in each image including 252 first-order statistics, 14 shape features, 336 gray-level co-occurrence matrix (GLCM), 224 gray-level run length matrix (GLRLM), 224 gray-level size zone matrix (GLSZM), 196 gray-level dependence matrix (GLDM), 70 neighboring gray-tone difference matrix (NGTDM). Since LE-CC and RC-CC images were analyzed, a total of 2632 radiomics features were extracted from each ROI (ITR, PTR5, PTR10, IPTR5, IPTR10).

The intraclass correlation coefficient (ICC) was used to evaluate the reproducibility of manual radiomics feature extraction. To calculate the intra- and inter-observer agreement of feature extraction, two radiologists (Radiologist 1 and Radiologist 2) firstly extracted the radiomics features using 40 randomly chosen patients to calculate the ICCs, respectively. Two weeks later, the procedure was repeated by Radiologist 1 and the remaining images were also analyzed by Radiologist 1. In this study, the radiomics features with ICCs of 0.80 or greater were selected and considered a mark of satisfactory intra- and inter-observer agreement.

### Feature selection and radiomics signature building

All feature selection processes were performed in the training cohort. Low variance was first used to filter features, the variance of each feature is calculated, and if it is below a threshold (0.80) we filter it out. Before performing low-variance filtering, all features were standardized using z-score normalization to ensure that the variance of each feature was comparable within the training cohort. Then, the least absolute shrinkage and selection operator (LASSO) logistic regression method was applied to select and identify the most stable and predictive features and to construct the radiomics signatures. To avoid over-fitting, the best parameter of the LASSO regularization parameter (α) was determined via 10-fold cross-validation. The radiomics signature score reflecting the respective equivocal HER2 (IHC2+) status for each patient was calculated through a linear combination of selected features weighted by their respective coefficients. On the basis of this procedure, five signatures were constructed from the five ROIs. The sensitivity and specificity values for evaluating the performance of the radiomics signatures in all cohorts were plotted to generate a receiver operator characteristic (ROC) curve, and the area under the curve (AUC) was calculated.

### Radiomics nomogram construction and comparison

**Figure 2:**
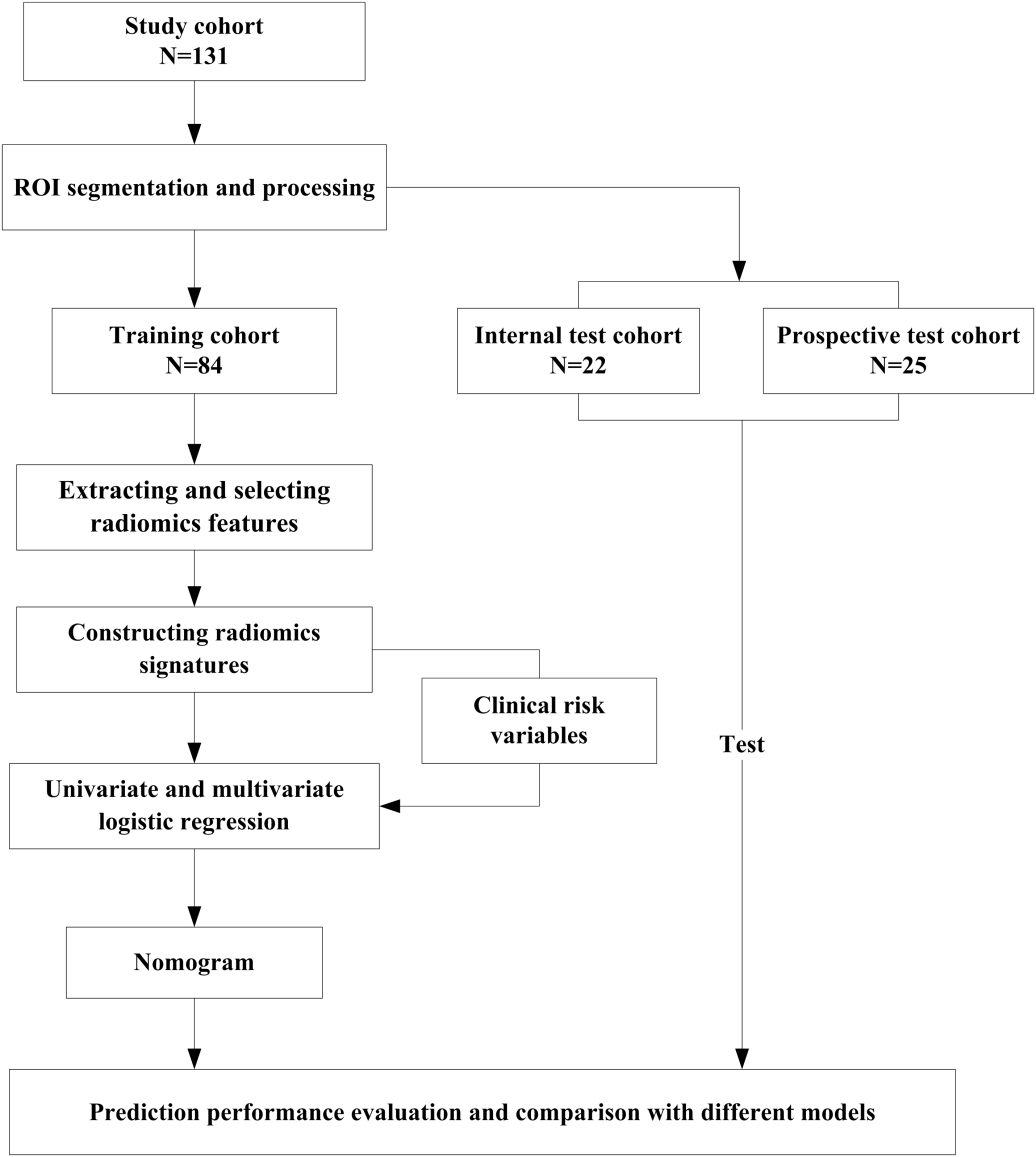
Study flow chart.

Firstly, the potential predictors among clinical variables and five radiomics signatures were identified using univariate logistic regression. Then these predictors were integrated into the multivariate logistic regression analysis with backward-stepwise selection based on minimal Akaike information criteria, which was used to select independent predictors of positive equivocal HER2 (IHC2+) status. A radiomics nomogram was established based on the independent predictors. For comparison, a clinical model was also built using only the selected clinical variables, and a radiomics model was built using only the selected radiomics signatures. To prove the generalization of the nomogram, a prospective cohort was used to test the models. The AUC, sensitivity, specificity, and accuracy were used to evaluate the predictive performance of the models. The study flow chart is shown in **Figure 2**.

### Statistical analysis

All statistical analyses were conducted with Python (version 3.6.6) and R software (version 4.0.3). Continuous variables were compared by two-sample t-test, while qualitative variables were analyzed by Chi-square test. LASSO regression and ROC curve analysis were conducted using the “LassoCV” function and “roc_curve” packages. The ROC curves were plotted to evaluate the performance of the models and the AUCs were calculated. The Youden index was chosen as the optimal cut-off value (31). Decision curve analysis (DCA) was employed to assess the net benefit of the models in a clinical context. The Hosmer-Lemeshow goodness of fit test was used to evaluate the calibration of the models and the calibration curve was plotted. DCA and calibration curve were performed using the “rmda” and “rms” packages. The DeLong test (32) was applied to compare the AUCs of different models. *P* < 0.05 was considered a statistically significant difference.

## Results

### Clinicopathological characteristics

A total of 131 patients were enrolled in this study. The training cohort included 84 patients (28 patients with positive IHC 2+ status, 56 patients with negative IHC 2+ status), the average age of patients was 54.2 ± 8.2 years old (range, 33–74 years old). The internal test cohort included 22 patients (8 patients with positive IHC 2+ status, 14 patients with negative IHC 2+ status), the average age of patients was 55.1 ± 8.9 years old (range, 38–75 years old). The prospective test cohort included 25 patients (10 patients with positive IHC 2+ status, 15 patients with negative IHC 2+ status), the average age of patients was 57.2 ± 11.1 years old (range, 32–80 years old). The results of the clinicopathological features between the patients with negative and positive equivocal HER2 (IHC 2+) status are listed in **Table 1**.

**Table 1:** Characteristics in the training, internal test, and prospective test cohorts.

Characteristics	Training cohort (n=84)			Internal test cohort (n=22)			Prospective test cohort (n=25)		
	HER2 2+ positive (n=28)	HER2 2+ negative (n=56)	*p*	HER2 2+ positive (n=8)	HER2 2+ negative (n=14)	*p*	HER2 2+ positive (n=10)	HER2 2+ negative (n=15)	*p*
Age, years (mean ± SD)	52.3 ± 8.8	54.9 ± 7.8	0.267	53.8 ± 4.8	55.8 ± 10.4	0.060	56.2 ± 10.8	57.8 ± 11.2	0.799
Diameter, cm (mean ± SD)	2.6 ± 1.1	3.1 ± 0.9	0.090	2.2 ± 0.7	3.5 ± 0.7	<0.010	3.7 ± 1.3	2.3 ± 0.9	0.001
ER			0.341			0.439			0.727
Negative	2	8		0	1		1	1	
Positive	26	48		8	13		9	14	
PR			1.000			0.674			0.105
Negative	6	12		1	1		4	2	
Positive	22	44		7	13		6	13	
Ki-67			0.701			0.339			<0.01
Negative	5	12		0	1		0	7	
Positive	23	44		8	13		10	8	

HER2, human epidermal growth factor receptor 2; ER, estrogen receptor; PR, progesterone receptor; SD, standard deviation.

### Performance of the radiomics signatures

**Figure 3:**
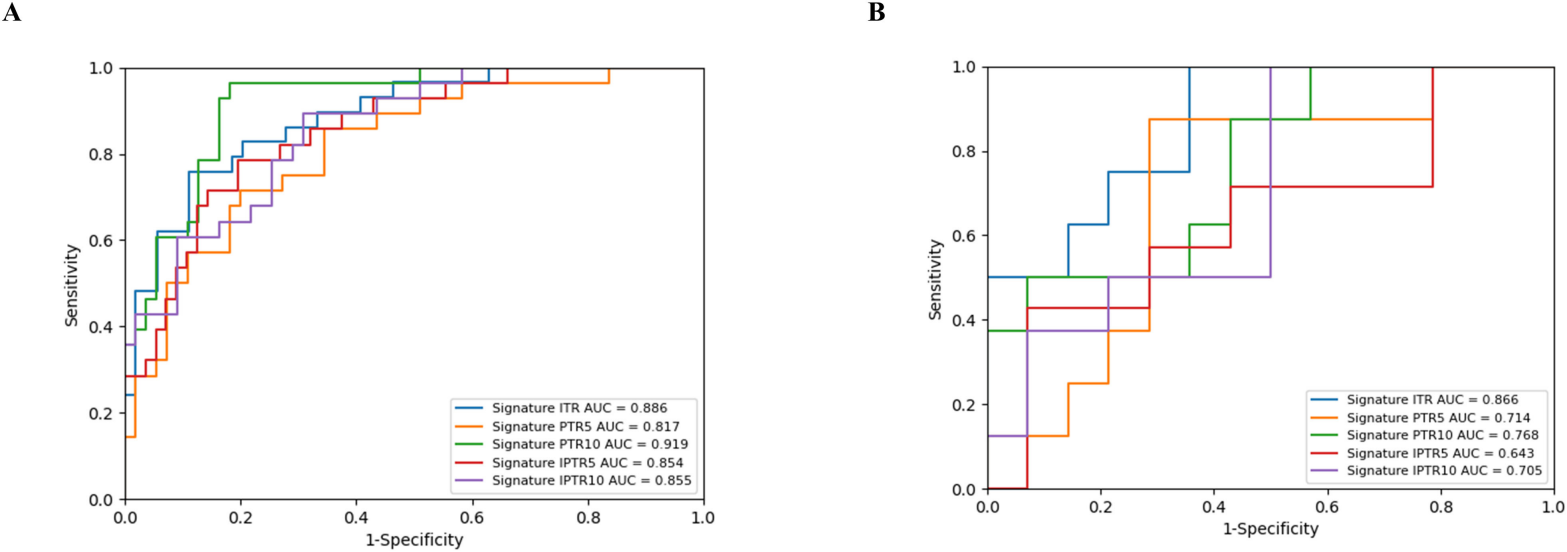
ROC curves of the five radiomics signatures for the prediction of equivocal HER2 (IHC 2+) status in the training cohort **(A)** and internal test cohort **(B)**.

The extracted features were identified as being highly reproducible based on the intra- and inter-observer ICCs ranging from 0.915 to 0.945 and 0.901 to 0.935, respectively. After selecting radiomics features, a total of 10, 8, 9, 7, and 6 features were selected as the most valuable features from ITR, PTR5, PTR10, IPTR5, and IPTR10, respectively, and then the five radiomics signatures were constructed. The detailed features and their respective coefficients are shown in **Supplementary Materials** (**Supplementary Table 1**). All five radiomics signatures showed encouraging results, with AUC values varying from 0.643 (95% confidence interval [CI], 0.364-0.921) to 0.866 (95% CI, 0.714-1.000) (**Figures 3A, B**). Among them, the radiomics signature based on ITR yielded the highest AUC value of 0.866 (95% CI, 0.714-1.000) in the internal test cohort. The detailed results are presented in **Supplementary Materials** (**Supplementary Table 2**).

### Performance of the nomogram, radiomics model, and clinical model

**Figure 4:**
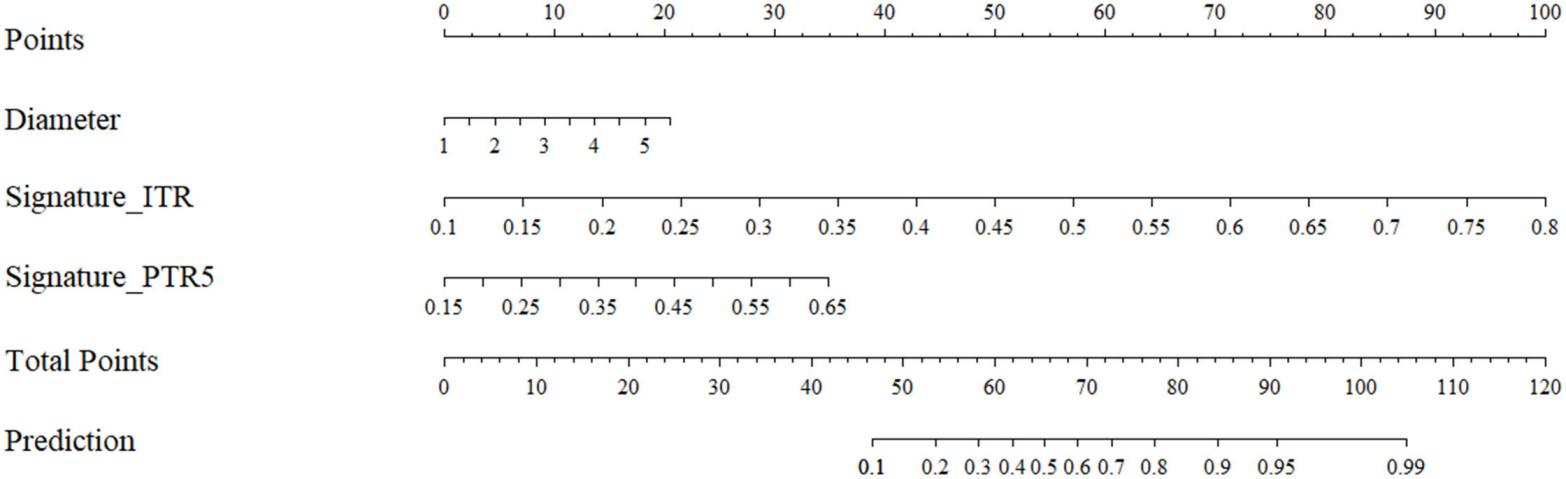
The nomogram for the prediction of equivocal HER2 (IHC 2+) status.

To ascertain the value of radiomics signatures and clinical variables, univariate and multivariate logistic regression analyses were conducted. Two radiomics signatures (constructed from ITR and PTR5) and the diameter were identified as significant predictors for identifying equivocal HER2 (IHC 2+) status. The detailed results are represented in **Table 2**. Then, the diameter, signature ITR and signature PTR5 were used to constructed the nomogram (**Figure 4**). In addition, the clinical model based on diameter and the radiomics model based on signature ITR and signature PTR5 were also constructed, respectively.

**Table 2:** Risk factors for predicting equivocal HER2 (IHC2+) status.

Variables	Univariate logistic regression		Multivariate logistic regression	
	OR (95% CI)	*p*	OR (95% CI)	*p*
Signature ITR	8.240(4.397-15.440)	<0.001*	8.600(3.868-19.123)	<0.001*
Signature PTR5	31.629(12.919-77.434)	<0.001*	3.490(1.062-11.467)	0.042*
Signature PTR10	3.259(2.104-5.046)	<0.001*	0.878(0.495-1.558)	0.658
Signature IPTR5	37.303(11.509-120.909)	<0.001*	0.857(0.204-3.599)	0.833
Signature IPTR10	29.807(7.724-115.025)	<0.001*	1.140(0.307-4.233)	0.845
Age	0.987(0.976-0.999)	0.044*	0.993(0.983-1.003)	0.630
Diameter	0.891(0.807-0.983)	0.024*	1.125(1.008-1.256)	0.039*

OR, odds ratio; CI, confidence interval; ITR, intratumoral region; PTR5, 5-mm peritumoral region; PTR10, 10-mm peritumoral region; IPTR5, intratumoral region + 5-mm peritumoral region); IPTR10, intratumoral region + 10-mm peritumoral region. *p < 0.05 was considered statistically significant.

**Figure 5:**
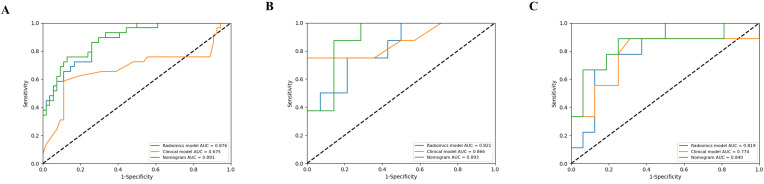
ROC curves of the models for the prediction of equivocal HER2 (IHC 2+) in the training **(A)**, internal test **(B)** and prospective test **(C)** cohorts.

**Figure 6:**
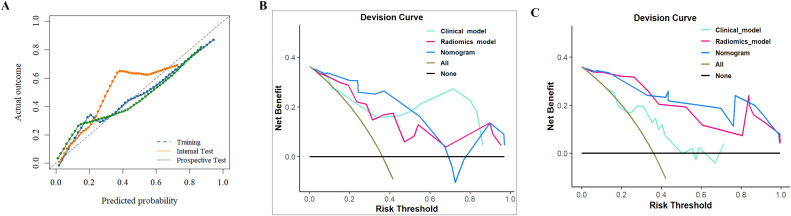
**(A)** Calibration curve of the nomogram in the three cohorts. The decision curve analysis of three models in the in the internal test **(B)** and prospective test **(C)** cohorts.

The decision threshold was determined based on the maximum Youden index in the training cohort. In this study, the decision threshold of the nomogram is 0.415. At this threshold, the predictive performance of the nomogram, such as AUC, sensitivity, specificity, accuracy, positive predictive value (PPV), and negative predictive value (NPV), across all cohorts is presented in the **Table 3**. Compared to other models, the nomogram achieved relatively good discrimination. In the internal test cohort, the AUC of the nomogram reached 0.893 (95CI%: 0.756-1.000), higher than the radiomics model with 0.821 (95CI%: 0.641-1.000) (Delong test, *P* = 0.292) and the clinical model with 0.866 (95CI%: 0.681-1.000) (Delong test, *P* = 0.314). In the prospective test cohort, the AUC of the nomogram was 0.840 (95CI%: 0.652-1.000), higher than the radiomics model with 0.819(95CI%: 0.649-0.999) (Delong test, *P* = 0.676) and the clinical model with 0.774 (95CI%: 0.547-1.000) (Delong test, *P* = 0.878). In the prospective test cohort, the nomogram achieved the highest sensitivity of 0.923. Detailed results are showed in **Figures 5A–C** and **Table 4**. The nomogram demonstrated good calibration, as evidenced by the calibration curves (**Figure 6A**) and further quantified by the Brier score. The Brier scores were 0.131 in the training cohort, 0.124 in the internal test cohort, and 0.156 in the prospective test cohort, indicating favorable predictive performance. **Figures 6B, C** showed the nomogram achieved a higher net benefit than the radiomics model and the clinical model in the internal test cohort.

**Table 3:** Predictive performance of nomogram.

Cohorts	AUC(95%CI)	SEN(95%CI)	SPE(95%CI)	ACC(95%CI)	PPV(95%CI)	NPV(95%CI)
Training cohort	0.891(0.823-0.959)	0.870(0.745-0.942)	0.759(0.561-0.890)	0.831(0.733- 0.905)	0.759(0.565-0.897)	0.870(0.755-0.941)
Internal test cohort	0.893(0.756-1.000)	0.857(0.561-0.974)	0.875(0.466-0.993)	0.864(0.651-0.971)	0.778(0.400-0.972)	0.923(0.663-0.990)
Prospective test cohort	0.840(0.652-1.000)	0.923(0.620-0.996)	0.667(0.354-0.887)	0.800(0.593-0.931)	0.667(0.349-0.901)	0.923(0.641-0.999)

AUC, area under curve; SEN, sensitivity; SPE, specificity; ACC, accuracy; PPV, positive predictive value; NPV, negative predictive value; CI, confidence interval.

**Table 4:** Comparison of radiomics model, clinical model, and nomogram prediction performance.

Models	AUC(95%CI)	SEN(95%CI)	SPE(95%CI)	ACC(95%CI)
Training cohort				
Radiomics model	0.876(0.802-0.950)	0.741(0.601-0.846)	0.862(0.674-0.955)	0.783(0.679- 0.866)
Clinical model	0.675(0.534-0.816)	0.889(0.767-0.954)	0.586(0.391-0.759)	0.781(0.679-0.866)
Nomogram	0.891(0.823-0.959)	0.870(0.745-0.942)	0.759(0.561-0.890)	0.831(0.733- 0.905)
Internal test cohort				
Radiomics model	0.821(0.641-1.000)	0.785(0.488-0.942)	0.750(0.355-0.955)	0.772(0.546-0.921)
Clinical model	0.866(0.681-1.000)	1.000(0.732-1.000)	0.750(0.355-0.955)	0.909(0.708-0.988)
Nomogram	0.893(0.756-1.000)	0.857(0.561-0.974)	0.875(0.466-0.993)	0.864(0.651-0.971)
Prospective test cohort				
Radiomics model	0.819(0.649-0.99)	0.866(0.584-0.976)	0.700(0.353-0.919)	0.800(0.593-0.931)
Clinical model	0.774(0.547-1.00)	0.846(0.536-0.973)	0.583(0.285-0.835)	0.720(0.506-0.879)
Nomogram	0.840(0.652-1.00)	0.923(0.620-0.996)	0.667(0.354-0.887)	0.800(0.593-0.931)

AUC, area under curve; SEN, sensitivity; SPE, specificity; ACC, accuracy; CI, confidence interval.

## Discussion

To achieve effective therapeutic efficacy for HER2-targeted treatment, precise equivocal HER2 (IHC 2+) status identification prior to treatment is indispensable. Although both IHC and FISH are commonly used approaches to determine equivocal HER2 (IHC 2+) status, their clinical applications are limited mainly by biopsy availability, procedure complexity and low reproducibility. In this study, we developed a nomogram using the selected radiomics signatures based on CEM and clinical risk factors to predict equivocal HER2 (IHC 2+) status, which achieved the highest AUC compared to the radiomic model and the clinical model. The results indicated that the nomogram yielded good discrimination and calibration.

Several studies have investigated that some abnormal imaging features, such as microcalcifications, breast density, or a spiculated mass on mammography are significantly associated with the HER2 status (33, 34). However, the performance of these features in predicting the HER2 status is limited and may be influenced by the subjective judgment of radiologists. Radiomics is an emerging field that can translate medical images into quantitative data for target task prediction. Multiple imaging methodologies, such as mammography and MRI, were revealed to be able to predict HER2 status for breast cancer based on radiomic features (17–19). However, they did not focus on equivocal HER2 (IHC 2+) status and CEM. At present, there is no study on the prediction of equivocal HER2 (IHC 2+) status based on CEM. To obtain more valuable radiomics features, this study extracted the radiomics features of LE-CC and RC-CC images to establish radiomics signatures, and integrated clinical risk factors to construct a nomogram, which achieved promising predictive performance. This demonstrates the value of CEM in predicting equivocal HER2 (IHC 2+) status of breast cancer.

Previous findings have shown that the peritumoral area can provide useful information to assist in the diagnosis of breast cancer, which is associated with lymphatic invasion and vascular infiltration (35, 36). Some researches demonstrated that radiomics features extracted from the peritumoral region could provide information different from those in the intratumoral region (24, 37, 38). In this study, five radiomics signatures were established based on different intratumoral and peritumoral regions. The signature ITR achieved the best predictive performance, followed by the PTR5 signature in the internal test cohort, indicating that peritumoral radiomics based on CEM could provide valuable information for predicting equivocal HER2 (IHC 2+) status. The results showed that PTR10 had a relatively lower predictive performance than PTR5, which may be related to the fact that a larger peritumoral area contains more fat or mammary glands and less tumor information.

Li et al (39) explored radiomics features of intratumoral and peritumoral regions on breast DCE-MRI to predict HER2 2+ status, and achieved a promising performance in the validation cohort (AUC = 0.840). However, it did not consider the role of clinical risk factors in the prediction task. In this study, the nomogram was developed based on radiomics features of intratumoral and peritumoral of CEM and clinical risk factor. Its predictive performance is superior to the single radiomics model or clinical model, indicating that both radiomics and clinical features have important value in predicting equivocal HER2 (IHC2+) status. Several recent studies have reported similar results, demonstrating the value of the nomogram established using radiomics signatures and clinical factors to evaluate pathological outcomes (40, 41). In addition, considering the clinical applicability of the nomogram, an independent prospective test cohort was enrolled to prove the generalization ability of the nomogram. Good predictive power was achieved, demonstrating that the nomogram has promising prospects for clinical application. While the nomogram demonstrated the highest AUC values, the wide confidence intervals and the non-significant results of the DeLong test when compared to the other models suggest that these findings require validation in larger, independent cohorts. This work represents a preliminary success in the pre-treatment prediction of equivocal HER2 (IHC2+) status using intra- and peritumoral radiomics, which could potentially assist in guiding personalized treatment.

However, our study had several limitations. First, this study is a single-center study with relatively inadequate sample size, and CEM examinations were performed using a single scanner type with a standardized protocol. Although it used the internal and prospective test cohorts to test the nomogram and achieved promising results. The generalizability and robustness of the nomogram to the images acquired from external test sets and different scanners or varying protocols remains unknown. In the future work, A larger sample size from different centers with different scanners or varying protocols is needed to further prove the generalization of the nomogram. Second, this study excluded non-mass lesions due to the difficulty in accurately delineating their boundaries on CEM, which may have introduced selection bias. The predictive performance of the proposed nomogram in non-mass breast cancer lesions therefore remains unclear, and further investigation is warranted to evaluate its applicability in the non-mass breast cancer lesions. Third, a manual segmentation method was employed in this study. Although favorable intra- and interobserver ICCs were obtained and an automated method was used for segmentation of peritumoral regions, which may have higher stability and be less time-consuming. Forth, predictive models based on radiomics features were developed only from images of the CC view. It is valuable to explore whether the extracting features in different views impact the performance of the prediction models, such as MLO views and the combination of CC and MLO views. Fifth, this study only incorporated CEM-based radiomics features without integrating multi-omics data such as genomics or transcriptomics. Currently, multi-omics studies have been conducted in other fields (42, 43). Future studies may combine multi-omics information to develop integrated models, thereby improving predictive performance and biological understanding of equivocal HER2 status.

## Conclusions

In conclusion, the nomogram combined with CEM-based intratumoral and peritumoral radiomics signatures and clinical variables could predict equivocal HER2(IHC2+) status noninvasively and conveniently. This may effectively guide the personalized treatment of patients with breast cancer in clinical practice.

## Data Availability

The original contributions presented in the study are included in the article/**Supplementary Material**. Further inquiries can be directed to the corresponding authors.

## References

[1] Litton JK Burstein HJ Turner NC . Molecular testing in breast cancer. Am Soc Clin Oncol Educ Book Am Soc Clin Oncol Annu Meeting. (2019) 39:e1-e7. doi: 10.1200/edbk_237715. PMID: 31099622

[2] Zhang S Wang H Zhang H Zhuang Q Zhu X Xiao Y . Clinicopathological and molecular features of HR (+) /HER2 (-) breast cancer patients with distinct endocrine resistance patterns. Chin J Cancer Res. (2025) 37:48–65. doi: 10.21147/j.issn.1000-9604.2025.01.04. PMID: 40078562 PMC11893345

[3] Piccart-Gebhart MJ Procter M Leyland-Jones B Goldhirsch A Untch M Smith I . Trastuzumab after adjuvant chemotherapy in HER2-positive breast cancer. N Engl J Med. (2005) 353:1659–72. doi: 10.1056/NEJMoa052306. PMID: 16236737

[4] Slamon D Eiermann W Robert N Pienkowski T Martin M Press M . Adjuvant trastuzumab in HER2-positive breast cancer. N Engl J Med. (2011) 365:1273–83. doi: 10.1056/NEJMoa0910383. PMID: 21991949 PMC3268553

[5] Waks AG Winer EP . Breast cancer treatment: a review. JAMA. (2019) 321:288–300. doi: 10.1001/jama.2018.19323. PMID: 30667505

[6] Rugo HS Chien AJ . HER2-positive breast cancer: is more treatment better? Lancet Oncol. (2016) 17:268–70. doi: 10.1016/S1470-2045(15)00623-3. PMID: 26874902

[7] Cameron D Piccart-Gebhart MJ Gelber RD Procter M Goldhirsch A de Azambuja E . 11 years' follow-up of trastuzumab after adjuvant chemotherapy in HER2-positive early breast cancer: final analysis of the HERceptin Adjuvant (HERA) trial. Lancet. (2017) 389:1195–205. doi: 10.1016/S0140-6736(16)32616-2. PMID: 28215665 PMC5465633

[8] Moelans CB de Weger RA Van der Wall E van Diest PJ . Current technologies for HER2 testing in breast cancer. Crit Rev Oncology/Hematology. (2011) 80:380–92. doi: 10.1016/j.critrevonc.2010.12.005. PMID: 21273092

[9] Rakha EA Pinder SE Bartlett JM Ibrahim M Starczynski J Carder PJ . Updated UK recommendations for HER2 assessment in breast cancer. J Clin Pathol. (2015) 68:93–9. doi: 10.1136/jclinpath-2014-202571. PMID: 25488926 PMC4316916

[10] Wolff AC Hammond MEH Allison KH Harvey BE Mangu PB Bartlett JMS . Human epidermal growth factor receptor 2 testing in breast cancer: American Society of Clinical Oncology/College of American Pathologists clinical practice guideline focused update. J Clin Oncol. (2018) 36:2105–22. doi: 10.1200/JCO.2018.77.8738. PMID: 29846122

[11] Lawson MB Herschorn SD Sprague BL Buist DSM Lee SJ Newell MS . Imaging surveillance options for individuals with a personal history of breast cancer: AJR expert panel narrative review. AJR Am J Roentgenology. (2022) 219:854–68. doi: 10.2214/ajr.22.27635. PMID: 35544374 PMC9691521

[12] Ghaderi KF Phillips J Perry H Lotfi P Mehta TS . Contrast-enhanced mammography: current applications and future directions. Radiographics. (2019) 39:1907–20. doi: 10.1148/rg.2019190079. PMID: 31697627

[13] Lee-Felker SA Tekchandani L Thomas M Gupta E Andrews-Tang D Roth A . Newly diagnosed breast cancer: comparison of contrast-enhanced spectral mammography and breast MR imaging in the evaluation of extent of disease. Radiology. (2017) 285:389–400. doi: 10.1148/radiol.2017161592. PMID: 28654337

[14] Sumkin JH Berg WA Carter GJ Bandos AI Chough DM Ganott MA . Diagnostic performance of MRI, molecular breast imaging, and contrast-enhanced mammography in women with newly diagnosed breast cancer. Radiology. (2019) 293:531–40. doi: 10.1148/radiol.2019190887. PMID: 31660801

[15] Lambin P Rios-Velazquez E Leijenaar R Carvalho S van Stiphout RG Granton P . Radiomics: extracting more information from medical images using advanced feature analysis. Eur J Cancer. (2012) 48:441–6. doi: 10.1016/j.ejca.2011.11.036. PMID: 22257792 PMC4533986

[16] Cao B Mi K Dai W Liu T Xie T Li Q . Prognostic and incremental value of computed tomography-based radiomics from tumor and nodal regions in esophageal squamous cell carcinoma. Chin J Cancer Res = Chung-Kuo Yen Cheng Yen Chiu. (2022) 34:71–82. doi: 10.21147/j.issn.1000-9604.2022.02.02. PMID: 35685995 PMC9086572

[17] Zhou J Tan H Li W Liu Z Wu Y Bai Y . Radiomics signatures based on multiparametric MRI for the preoperative prediction of the HER2 status of patients with breast cancer. Acad Radiol. (2021) 28:1352–60. doi: 10.1016/j.acra.2020.05.040. PMID: 32709582

[18] Bitencourt AGV Gibbs P Rossi Saccarelli C Daimiel I Lo Gullo R Fox MJ . MRI-based machine learning radiomics can predict HER2 expression level and pathologic response after neoadjuvant therapy in HER2 overexpressing breast cancer. EBioMedicine. (2020) 61:103042. doi: 10.1016/j.ebiom.2020.103042. PMID: 33039708 PMC7648120

[19] Zhou J Tan H Bai Y Li J Lu Q Chen R . Evaluating the HER-2 status of breast cancer using mammography radiomics features. Eur J Radiol. (2019) 121:108718. doi: 10.1016/j.ejrad.2019.108718. PMID: 31711023

[20] Niu S Jiang W Zhao N Jiang T Dong Y Luo Y . Intra- and peritumoral radiomics on assessment of breast cancer molecular subtypes based on mammography and MRI. J Cancer Res Clin Oncol. (2022) 148:97–106. doi: 10.1007/s00432-021-03822-0. PMID: 34623517 PMC11800953

[21] Mao N Shi Y Lian C Wang Z Zhang K Xie H . Intratumoral and peritumoral radiomics for preoperative prediction of neoadjuvant chemotherapy effect in breast cancer based on contrast-enhanced spectral mammography. Eur Radio. (2022) 32:3207–19. doi: 10.1007/s00330-021-08414-7. PMID: 35066632

[22] Wang S Sun Y Li R Mao N Li Q Jiang T . Diagnostic performance of perilesional radiomics analysis of contrast-enhanced mammography for the differentiation of benign and Malignant breast lesions. Eur Radiol. (2022) 32:639–49. doi: 10.1007/s00330-021-08134-y. PMID: 34189600

[23] Salgado R Denkert C Campbell C Savas P Nuciforo P Aura C . Tumor-infiltrating lymphocytes and associations with pathological complete response and event-free survival in HER2-positive early-stage breast cancer treated with lapatinib and trastuzumab: a secondary analysis of the NeoALTTO trial. JAMA Oncol. (2015) 1:448–54. doi: 10.1001/jamaoncol.2015.0830. PMID: 26181252 PMC5551492

[24] Braman N Prasanna P Whitney J Singh S Beig N Etesami M . Association of peritumoral radiomics with tumor biology and pathologic response to preoperative targeted therapy for HER2 (ERBB2)-positive breast cancer. JAMA Netw Open. (2019) 2:e192561. doi: 10.1001/jamanetworkopen.2019.2561. PMID: 31002322 PMC6481453

[25] Hsu H Lee KH Karmakar R Mukundan A Attar RS Liu PH . From innovation to application: can emerging imaging techniques transform breast cancer diagnosis? Diagnostics (Basel). (2025). doi: 10.3390/diagnostics15212718. PMID: 41226009 PMC12610140

[26] Leung JH Karmakar R Mukundan A Thongsit P Chen MM Chang WY . Systematic meta-analysis of computer-aided detection of breast cancer using hyperspectral imaging. Bioengineering (Basel). (2024). doi: 10.3390/bioengineering11111060. PMID: 39593720 PMC11591395

[27] Bhimani C Matta D Roth RG Liao L Tinney E Brill K . Contrast-enhanced spectral mammography: technique, indications, and clinical applications. Acad Radiol. (2017) 24:84–8. doi: 10.1016/j.acra.2016.08.019. PMID: 27773458

[28] Allison KH Hammond MEH Dowsett M McKernin SE Carey LA Fitzgibbons PL . Estrogen and progesterone receptor testing in breast cancer: ASCO/CAP guideline update. J Clin Oncol. (2020) 38:1346–66. doi: 10.1200/JCO.19.02309. PMID: 31928404

[29] Nielsen TO Leung SCY Rimm DL Dodson A Acs B Badve S . Assessment of Ki67 in breast cancer: updated recommendations from the International Ki67 in Breast Cancer Working Group. J Natl Cancer Inst. (2021) 113:808–19. doi: 10.1093/jnci/djaa201. PMID: 33369635 PMC8487652

[30] van Griethuysen JJM Fedorov A Parmar C Hosny A Aucoin N Narayan V . Computational radiomics system to decode the radiographic phenotype. Cancer Res. (2017) 77:e104-e107. doi: 10.1158/0008-5472.Can-17-0339. PMID: 29092951 PMC5672828

[31] Marcus D Ruopp NJP Brian W Whitcomb BW Schisterman EF . Youden index and optimal cut-point estimated from observations affected by a lower limit of detection. Biom J. (2008) 50:419–30. doi: 10.1002/bimj.200710415. PMID: 18435502 PMC2515362

[32] R DE M DD L C-P . Comparing the areas under two or more correlated receiver operating characteristic curves: a nonparametric approach. Biometrics. (1988) 44:837–45. 3203132

[33] Li E Guida JL Tian Y Sung H Koka H Li M . Associations between mammographic density and tumor characteristics in Chinese women with breast cancer. Breast Cancer Res Treat. (2019) 177:527–36. doi: 10.1007/s10549-019-05325-6. PMID: 31254158 PMC7304859

[34] Shin HJ Kim HH Huh MO Kim MJ Yi A Kim H . Correlation between mammographic and sonographic findings and prognostic factors in patients with node-negative invasive breast cancer. Br J Radiol. (2011) 84:19–30. doi: 10.1259/bjr/92960562. PMID: 20682592 PMC3473801

[35] Schoppmann SF Bayer G Aumayr K Taucher S Geleff S Rudas M . Prognostic value of lymphangiogenesis and lymphovascular invasion in invasive breast cancer. Ann Surg. (2004) 240:306–12. doi: 10.1097/01.sla.0000133355.48672.22. PMID: 15273556 PMC1356408

[36] Ejlertsen B Jensen MB Rank F Rasmussen BB Christiansen P Kroman N . Population-based study of peritumoral lymphovascular invasion and outcome among patients with operable breast cancer. J Natl Cancer Inst. (2009) 101:729–35. doi: 10.1093/jnci/djp090. PMID: 19436035

[37] Zhou J Zhang Y Chang KT Lee KE Wang O Li J . Diagnosis of benign and Malignant breast lesions on DCE-MRI by using radiomics and deep learning with consideration of peritumor tissue. J Magn Reson Imaging. (2020) 51:798–809. doi: 10.1002/jmri.26981. PMID: 31675151 PMC7709823

[38] Wu J Li B Sun X Cao G Rubin DL Napel S . Heterogeneous enhancement patterns of tumor-adjacent parenchyma at MR imaging are associated with dysregulated signaling pathways and poor survival in breast cancer. Radiology. (2017) 285:401–13. doi: 10.1148/radiol.2017162823. PMID: 28708462 PMC5673053

[39] Li C Song L Yin J . Intratumoral and peritumoral radiomics based on functional parametric maps from breast DCE-MRI for prediction of HER-2 and Ki-67 status. J Magn Reson Imaging. (2021) 54:703–14. doi: 10.1002/jmri.27651. PMID: 33955619

[40] Yu Y Tan Y Xie C Hu Q Ouyang J Chen Y . Development and validation of a preoperative magnetic resonance imaging radiomics-based signature to predict axillary lymph node metastasis and disease-free survival in patients with early-stage breast cancer. JAMA Netw Open. (2020) 3:e2028086. doi: 10.1001/jamanetworkopen.2020.28086. PMID: 33289845 PMC7724560

[41] Tang TY Li X Zhang Q Guo CX Zhang XZ Lao MY . Development of a novel multiparametric MRI radiomic nomogram for preoperative evaluation of early recurrence in resectable pancreatic cancer. J Magn Reson Imaging. (2020) 52:231–45. doi: 10.1002/jmri.27024. PMID: 31867839 PMC7317738

[42] Wang L Zhang X Zhang J Liu J Chen Y Huang W . Multiomics machine learning to predict neoadjuvant chemotherapy outcome and relapse of breast cancer. BME Front. (2026) 7:212. doi: 10.34133/bmef.0212. PMID: 41608269 PMC12835490

[43] He Y Xie J Zhong S Zhan C Dai F Lai H . A deep learning-generated mixed tumor-stroma ratio for prognostic stratification and multi-omics profiling in bladder cancer. Res (Wash D C). (2026) 9:1053. doi: 10.34133/research.1053. PMID: 41602481 PMC12833823

