# Supplementary material for: Intra- and peritumoral radiomics for predicting equivocal HER2 status of breast cancer on contrast-enhanced mammography
Source: Front Oncol. 2026 Apr 29;16:1807530. doi: 10.3389/fonc.2026.1807530 (PMC13167432; doi:10.3389/fonc.2026.1807530)
Supplement: Supplementary file 1 [file Table1.docx]

# Supplementary Materials

**Table S1.** The selected radiomics features and the LASSO coefficient of five ROIs

| **ROI** | **Radiomics Features** | **Modality** | **Coefficient** |
| --- | --- | --- | --- |
| **ITR** | original_shape_Elongation | Low-energy | 0.018491 |
|  | wavelet-HLL_glszm_GrayLevelVariance | Low-energy | 0.035523 |
|  | wavelet-HLL_gldm_SmallDependenceLowGrayLevelEmphasis | Low-energy | 0.035485 |
|  | wavelet-LHH_glszm_SmallAreaLowGrayLevelEmphasis | Low-energy | -0.023355 |
|  | wavelet-LHH_gldm_LargeDependenceHighGrayLevelEmphasis | Low-energy | -0.015774 |
|  | wavelet-LHL_glrlm_LongRunHighGrayLevelEmphasis | Low-energy | -0.064697 |
|  | wavelet-LHL_firstorder_Median | Low-energy | -0.000887 |
|  | gradient_firstorder_MeanAbsoluteDeviation | Low-energy | -0.000565 |
|  | wavelet-HHL_glrlm_ShortRunLowGrayLevelEmphasis | Recombined | 0.011011 |
|  | wavelet-HHL_glrlm_ShortRunEmphasis | Recombined | 0.015776 |
| **PTR5** | wavelet-HHL_glcm_SumSquares | Recombined | -2.123360e-02 |
|  | wavelet-HLL_glszm_SmallAreaHighGrayLevelEmphasis | Recombined | -1.708737e-02 |
|  | wavelet-HHH_glszm_GrayLevelVariance | Recombined | 5.465841e-02 |
|  | wavelet-HHH_glszm_GrayLevelNonUniformityNormalized | Recombined | 8.284966e-10 |
|  | original_shape_Elongation | Low-energy | 1.412578e-02 |
|  | original_shape_MajorAxisLength | Low-energy | -1.303178e-02 |
|  | wavelet-HLH_glszm_SizeZoneNonUniformity | Low-energy | -1.505199e-02 |
|  | wavelet-HHL_glcm_SumSquares | Low-energy | -4.054199e-02 |
| **PTR10** | square_glrlm_RunEntropy | Low-energy | -3.253634e-02 |
|  | wavelet-HHH_glszm_HighGrayLevelZoneEmphasis | Low-energy | 1.044741e-01 |
|  | square_gldm_DependenceEntropy | Low-energy | -1.940484e-02 |
|  | gradient_gldm_DependenceEntropy | Low-energy | -5.350472e-17 |
|  | exponential_gldm_DependenceEntropy | Low-energy | -2.675236e-18 |
|  | original_shape_Elongation | Recombined | 9.849506e-02 |
|  | logarithm_glrlm_RunEntropy | Recombined | -1.098314e-02 |
|  | wavelet-HLH_glszm_GrayLevelNonUniformity | Low-energy | -4.965033e-02 |
|  | wavelet-HHH_glszm_ZoneVariance | Low-energy | -5.477850e-02 |
| **IPTR5** | original_shape_Elongation | Recombined | 0.019154 |
|  | wavelet-HLL_glszm_SizeZoneNonUniformity | Recombined | -0.017246 |
|  | original_shape_Maximum2DDiameterRow | Low-energy | -0.000326 |
|  | original_shape_Elongation | Low-energy | 0.003004 |
|  | wavelet-HLH_glrlm_LongRunHighGrayLevelEmphasis | Low-energy | -0.010730 |
|  | wavelet-HHH_glrlm_ShortRunHighGrayLevelEmphasis | Low-energy | 0.049855 |
|  | wavelet-HHH_gldm_DependenceNonUniformityNormalized | Low-energy | -0.027965 |
| **IPTR10** | original_shape_Elongation | Recombined | 1.343478e-02 |
|  | original_shape_Maximum2DDiameterRow | Low-energy | -3.306736e-02 |
|  | wavelet-HLH_glrlm_LongRunHighGrayLevelEmphasis | Low-energy | -1.228527e-02 |
|  | wavelet-HLL_glrlm_GrayLevelVariance | Low-energy | 2.796438e-03 |
|  | wavelet-HLL_glrlm_GrayLevelNonUniformityNormalized | Low-energy | -6.266809e-07 |
|  | wavelet-HHH_glszm_ZonePercentage | Low-energy | 3.325324e-02 |

Note: ROI, region of interest; ITR, intratumoral region; PTR5, 5-mm peritumoral region; PTR10, 10-mm peritumoral region; IPTR5, intratumoral region + 5-mm peritumoral region); IPTR10, intratumoral region + 10-mm peritumoral region

Table S2: Performance of five signatures in the training and internal test cohort

|  | **AUC(95%CI)** | **SEN(95%CI)** | **SPE(95%CI)** | **ACC(95%CI)** |
| --- | --- | --- | --- | --- |
| **Training cohort** | | | | |
| Signature ITR | 0.886(0.813-0.959) | 0.888(0.766-0.954) | 0.758(0.560-0.889) | 0.843(0.747- 0.913) |
| Signature PTR5 | 0.919(0.861-0.977) | 0.818(0.686-0.904) | 0.964(0.797-0.998) | 0.867(0.775-0.931) |
| Signature PTR10 | 0.817(0.721-0.912) | 0.800(0.666-0.891) | 0.714(0.511-0.860) | 0.771(0.665- 0.856) |
| Signature IPTR5 | 0.855(0.773-0.935) | 0.690(0.550-0.804) | 0.892(0.706-0.971) | 0.759(0.665-0.846) |
| Signature IPTR10 | 0.854(0.771-0.936) | 0.803(0.671-0.893) | 0.785(0.585-0.909) | 0.797(0.695-0.877) |
| **Internal test cohort** | | | | |
| Signature ITR | 0.866(0.714-1.000) | 0.642(0.356-0.860) | 1.000(0.597-1.000) | 0.772(0.546-0.921) |
| Signature PTR5 | 0.768(0.557-0.978) | 0.571(0.296-0.811) | 0.875(0.466-0.953) | 0.681(0.451-0.861) |
| Signature PTR10 | 0.714(0.479-0.949) | 0.714(0.420-0.904) | 0.875(0.466-0.993) | 0.772(0.546-0.921) |
| Signature IPTR5 | 0.705(0.475-0.935) | 0.500(0.240-0.759) | 1.000(0.597-1.000) | 0.681(0.451-0.861) |
| Signature IPTR10 | 0.643(0.364-0.921) | 0.928(0.641-0.996) | 0.428(0.118-0.797) | 0.761(0.528-0.917) |

Note: AUC, area under curve; SEN, sensitivity; SPE, specificity; ACC, accuracy; CI, confidence interval; ITR, intratumoral region; PTR5, 5-mm peritumoral region; PTR10, 10-mm peritumoral region; IPTR5, intratumoral region + 5-mm peritumoral region); IPTR10, intratumoral region + 10-mm peritumoral region
